# Efficacy and safety of Kangfuxin liquid combined with aminosalicylic acid for the treatment of ulcerative colitis: A systematic review and meta-analysis

**DOI:** 10.1097/MD.0000000000010807

**Published:** 2018-05-25

**Authors:** Hui-biao Li, Mu-yuan Chen, Zhen-wen Qiu, Qing-qun Cai, De-tang Li, Hong-mei Tang, Xin-lin Chen

**Affiliations:** a The First Clinical College, The First Affiliated Hospital; b School of Basic Medical Science, Guangzhou University of Chinese Medicine, Guangzhou, Guangdong, China.

**Keywords:** aminosalicylic acid, Kangfuxin liquid, meta-analysis, ulcerative colitis

## Abstract

**Background:**

To systematically evaluate the clinical efficacy and safety of Kangfuxin liquid (KFXL) combined with aminosalicylic acid (ASA) in treating ulcerative colitis (UC).

**Methods:**

The PubMed, Cochrane Library, Embase, CBM, Wan fang, the Chinese Scientific Journal Database (VIP), and Chinese National Knowledge Infrastructure (CNKI) databases were systematically searched for randomized controlled trials of KFXL combined with ASA for UC from the inception dates to March 3, 2017. Two researchers independently screened the literature, extracted data, and evaluated the methodological quality according to the inclusion criteria. The meta-analysis was performed using Review Manager software (RevMan, Version 5.3, Copenhagen: The Nordic Cochrane Centre, The Cochrane Collaboration, 2014), and the risk of bias was assessed using the Cochrane Collaboration Tool.

**Results:**

A total of 39 randomized controlled trials (RCTs) involving 3204 patients fulfilled the inclusion criteria. Compared with ASA alone, KFXL combined with ASA significantly improved the clinical effectiveness rate [RR = 1.19, 95% CI: (1.16, 1.23), *P < *.00001], reduced the relapse rate [RR = 0.26, 95% CI: (0.18, 0.38), *P < *.00001], reduced the inflammation factor levels of TNF-a, IL-1, IL-6, IL-8, and C-reactive protein, reduced the coagulation index of fibrinogen, increased the coagulation index of prothrombin time, and mean platelet volume, and reduced the clinical symptoms of abdominal pain, diarrhoea, pus and bloody stool, and tenesmus. However, KFXL combined with ASA did not increase the adverse event incidence [RR = 0.74, 95% CI (0.42, 1.32), *P* = .31], and no severe adverse events were reported.

**Conclusion:**

KFXL combined with ASA has good therapeutic effect for UC and might be a safe approach in managing UC. More high-quality, multicenter randomized, double-blind trials with a large sample size are required to generate a high level of clinical evidence.

## Abstract 2

Supplemental Digital Content is available in the text

## 1 Introduction

Ulcerative colitis (UC) is a chronic nonspecific inflammatory disease caused by immune abnormalities, mental disorders, genetics, and other factors. Its main clinical manifestations are abdominal pain, diarrhoea, bloody stool, weight loss, etc.^[1]^ According to an epidemiological survey, the highest incidence rates of UC in Europe, Asia, and North America were 24.3/10 million, 6.3/10 million, and 19.2/10 million, respectively, and the highest prevalence rates were 505/10 million, 63.6/10 million, and 249/10 million, respectively.^[2]^ The incidence rates of UC in Asia, Latin America, South Africa and other developing countries and regions are increasing year by year. UC has become one of the most common diseases in the world.^[3,4]^ UC seriously affects human health and quality of life because of its long duration and recurrent attacks, and it has the risk of developing into colorectal cancer.^[5]^

In recent years, a large number of clinical studies have shown that KFXL combined with ASA has a good effect in the treatment of UC. KFXL is a Chinese medicine extracted from *Periplaneta americana* dried worms. The main components of the drug are polyhydric alcohols, peptides, mucin, amino acids and other active substances, with the functions of acid suppression, anti-inflammation, improvement of gastrointestinal mucosal microcirculation, promotion of granulation tissue hyperplasia, acceleration of diseased tissue regeneration, and improvement of immunity.^[6,7]^ Pharmacological studies have found that KFXL can inhibit the expression of MMP-3 and MMP-13, decrease the levels of NF-κB, IL-1β, TNF-α, and INF-γ, increase the level of IL-4, and upregulate the expression of EGF and HGF in colonic mucosa to achieve the purpose of treating UC.^[8–12]^

However, no meta-analysis has been conducted to summarize these research studies to determine whether KFXL combined with ASA is more efficacious than ASA alone in the treatment of UC. To provide more evidence for clinical decision making, we collected published studies covering RCTs of KFXL combined with ASA vs ASA alone in the treatment of UC and conducted a meta-analysis to assess its efficacy and safety.

## 2 Methods

### 2.1 Information sources and search strategies

A computerized search of the PubMed, Embase, Medline, Cochrane Library, the Chinese National Knowledge Infrastructure (CNKI), the Chinese Scientific Journal Database (VIP), the Chinese Biomedical Literature Database (CBM), and the Wanfang databases were conducted from inception to March 3, 2017. There was no restriction on language or publication status. The search terms for literature searching were as follows: “Kangfuxin,” “Kangfuxin liquid,” or “Kangfuxin Ye”; “ulcerative colitis”; and “randomized controlled trial,” “controlled clinical trial,” “random,” “randomly,” “randomized” or “control.” To collect sufficient trials, the reference lists of retrieved articles were also reviewed.

### 2.2 Inclusion criteria

We conducted this study according to the preferred reporting items for systematic reviews and meta-analysis (PRISMA statement).^[13]^

Studies were included for analysis if they satisfied the following criteria. *Participants*: all participants enrolled in this study were diagnosed as UC.^[14–20]^ No limitations on gender, age, or ethnicity of the participants were set. *Type of design:* RCTs were included, regardless of blinding. Animal studies were not considered. *Type of intervention*: KFXL combined with ASA was chosen for the treatment group and ASA for the control group. The ASA used in the treatment groups should be the same as the controls in the category, dosage and method of administration. If other co-interventions such as another herbal formula, cupping, Tai Chi, moxibustion, acupuncture, qigong, massage, yoga, and aromatherapy were used in either the treatment group or the control group, those studies were excluded. *Type of outcome:* outcomes included at least the total clinical effectiveness rate or other indices of clinical improvement. When several trials from the same authors were identified as duplicates, we only included the most recent trial with the largest number of patients or longer follow-up. There were no language or publication status restrictions.

### 2.3 Data extraction

Two of the 3 investigators (HL, MC or DL) independently screened all the titles and abstracts of the eligible studies. The following information from primary trials was extracted: first author name, year of publication, age, gender, number of patients, details of interventions, co-interventions, outcomes, the duration of treatment, and adverse effects. Disagreements were resolved through discussion or from a third partner.

### 2.4 Assessment of risk of bias

Two reviewers (HL, MC) independently evaluated the risk of bias of each study using the assessment tool from the Cochrane Handbook.^[21]^ The criteria consisted of the following 7 items: sequence generation, allocation concealment, blinding of participants and personnel, blinding of outcome assessments, incomplete outcome data, selective reporting, and other sources of bias. A judgement of “low” indicated low risk of bias, “high” indicated high risk of bias, and “Unclear” indicated unclear risk of bias. The disagreements in data collection were discussed with a third author (DL) and resolved by a consensus process.

### 2.5 Data analysis

The meta-analysis was performed using the Review Manager 5.3 software. Risk ratio (RR) and 95% confidence interval (CI) were calculated for dichotomous data. For continuous data, standardized mean difference (SMD) and 95% CI were calculated. If different measurement indices that adopted different tools were used in the various studies, SMD was preferred over the weighted mean difference. The heterogeneity among the trials was identified by χ^2^, using Cochrane Handbook *Q* test and quantified by *I*^2^, which determines the per cent of the total variability that cannot be ascribed to chance. A fixed-effects model was used when there was no significant heterogeneity (*P* > .05, *I*^2^ < 50%). Otherwise, a random-effects model was applied (*P < *.05, *I*^2^>50%). Subgroup analyses were carried out based on the doses and medicines. Publication bias was assessed by funnel plot analysis if the group included more than 10 trials. When possible, sensitivity analyses were conducted for all outcomes.

## 3 Results

### 3.1 Study identification

**Figure 1:**
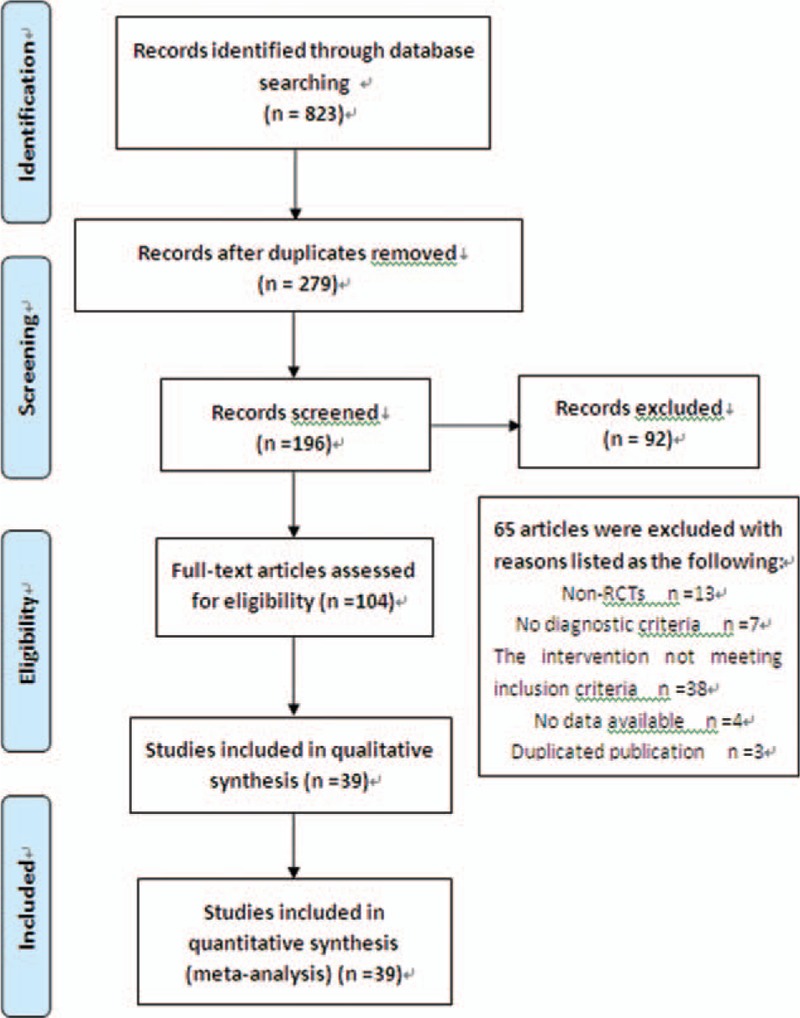
Flow diagram of study selection and identification.

A total of 823 potentially relevant articles were initially screened in the 7 electronic databases based on our literature search strategy. After removing 544 duplicates, 279 articles were identified for further analysis. The titles and abstracts of the remaining articles were read by the reviewers, and 92 articles that did not meet the inclusion criteria were excluded. Next, 104 articles were checked for the full texts, and 65 articles were excluded. Finally, 39 trials were included for further appraisal and data extraction.^[22–60]^ A flowchart shows the process of study selection and identification (Fig. 1).

### 3.2 Study characteristics

All the eligible trials were based on randomized controlled trials. A total of 3204 UC patients were enrolled, with 1622 in the treatment group and 1582 in the control group. The included trials were published as the full text from 2006 to 2017. All of these trials were carried out in China, and all the participants involved were Chinese. The number of patients in the intervention group varied from 36 to 268. The duration of the treatment ranged from 14 days to 60 days. All the studies used a two-arm design (one treatment group vs one control group). For interventions, patients in the control group received ASA, including 5-ASA (n = 28),^[22–49]^ SASP (n = 10),^[50–59]^ and OSLS (n = 1).^[60]^ Patients in the treatment groups were treated with KFXL on the basis of the control group. The basic characteristics of the 39 included randomized trials are summarized in Table 1.

**Table 1:**
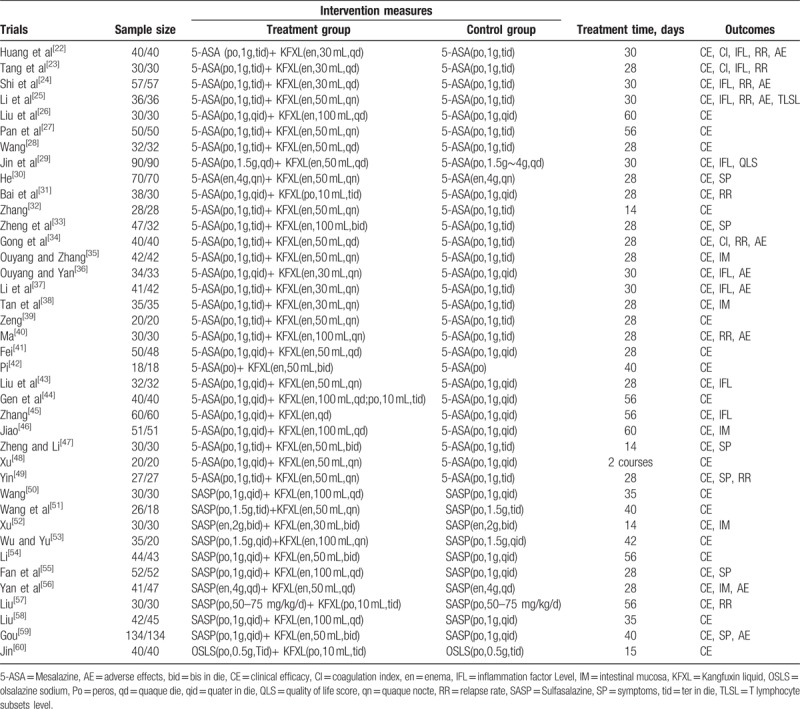
The characteristic of the eligible trials.

### 3.3 Risk of bias

**Figure 2:**
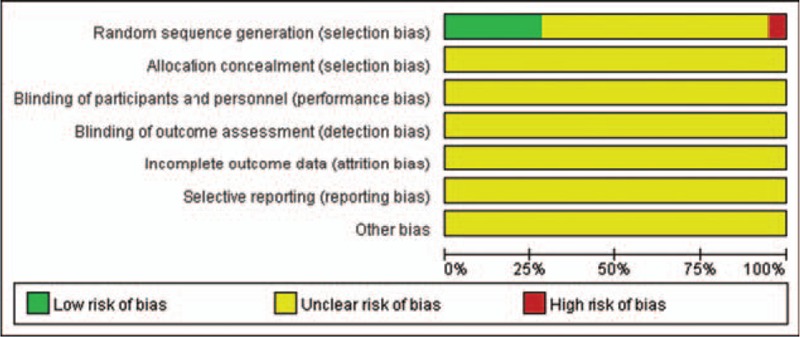
Risk of bias graph.

**Figure 3:**
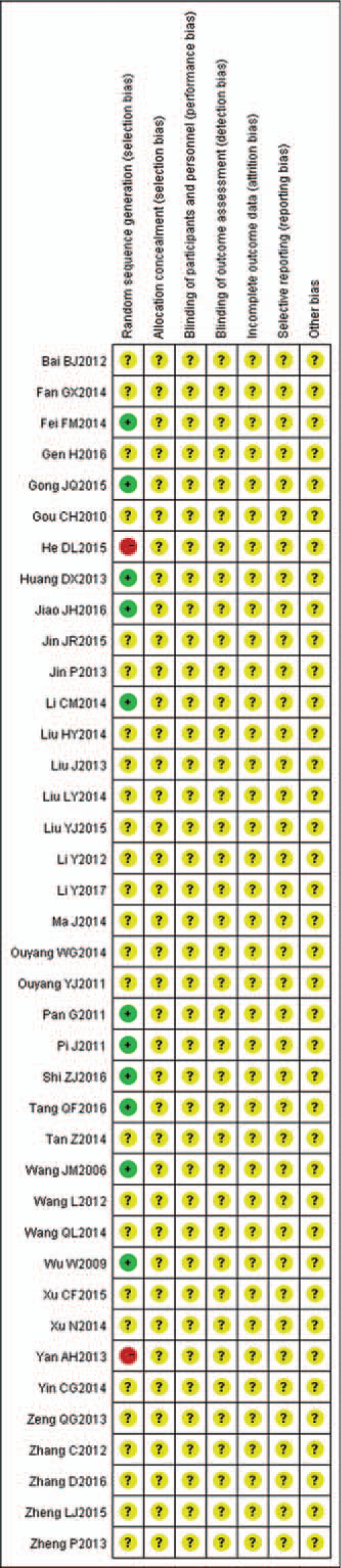
Risk of bias summary.

All the trials mentioned random allocation. Only 11 trials^[22–24,27,34,41,42,46,51,53,54]^ described the method of randomization (random number table), and 2 trials^[30,56]^ were randomized according to the order of visits, which means a high risk of bias; the other trials did not mention any information about randomization methods. All the trials did not state the method of allocation concealment and blinding. Incomplete outcome data, selective outcome reporting, and other sources of bias were assessed as unclear risk of bias in all of the trials. The risk of bias in all the trials was considered to have a “high risk of bias.” The details of the risk of bias of each trial are presented in Figures 2 and 3.

### 3.4 Clinical remission rate

Thirty-nine studies^[22–60]^ in UC patients were compared with respect to the primary outcome of clinical remission. There was no significant heterogeneity for the clinical remission rate between the 2 groups (*P = *.31, *I*^2^ = 9%). The meta-analysis was performed using a fixed-effects model. The results showed that the clinical remission of KFXL combined with ASA treatment improved significantly compared with ASA treatment (*P < *.00001), with a RR of 1.19 and 95% CI (1.16, 1.23).

### 3.5 Subgroup analysis of different medicines

**Figure 4:**
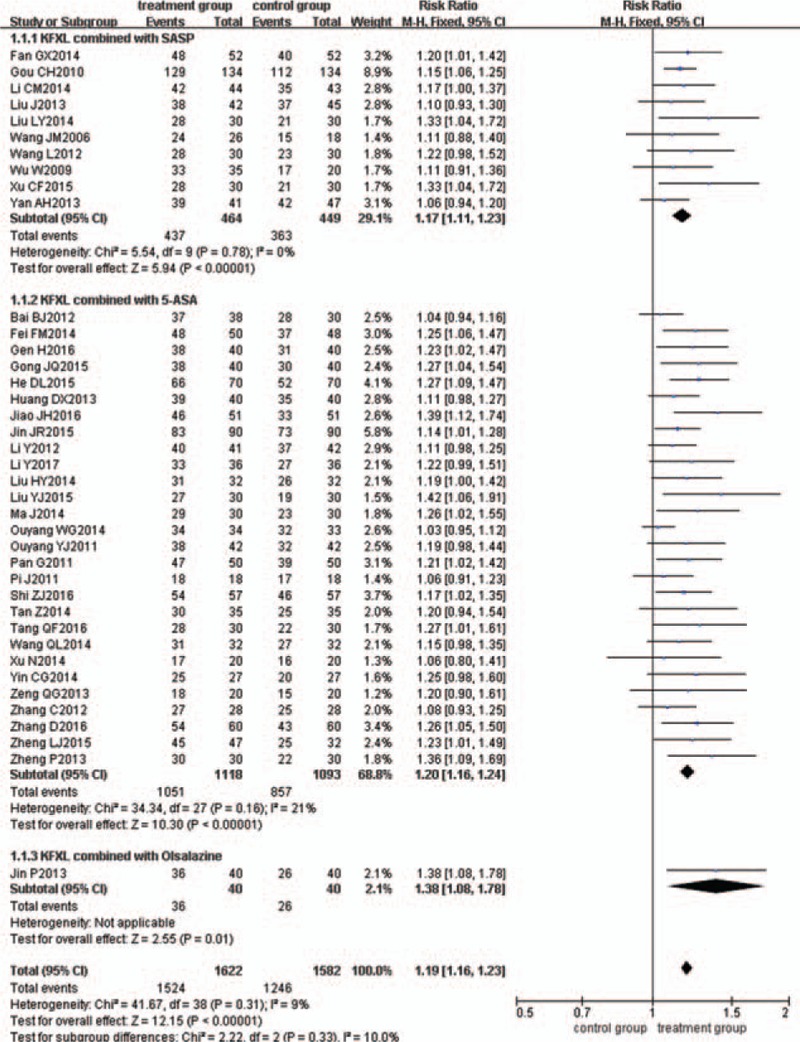
Meta-analysis of the clinical remission rate for the subgroup analysis of different medicines.

Subgroup analysis was used to evaluate the efficacy of different medicines. Compared with ASA alone, KFXL plus SASP, and KFXL plus 5-ASA both had significant improvements in clinical remission, with RR = 1.17 (95% CI = 1.11, 1.23, n = 10), and RR = 1.20 (95% CI = 1.16, 1.24, n = 28), respectively (Fig. 4). This finding indicates that KFXL combined with ASA may have better potential clinical efficacy than ASA used alone.

### 3.6 Subgroup analysis of different doses

**Figure 5:**
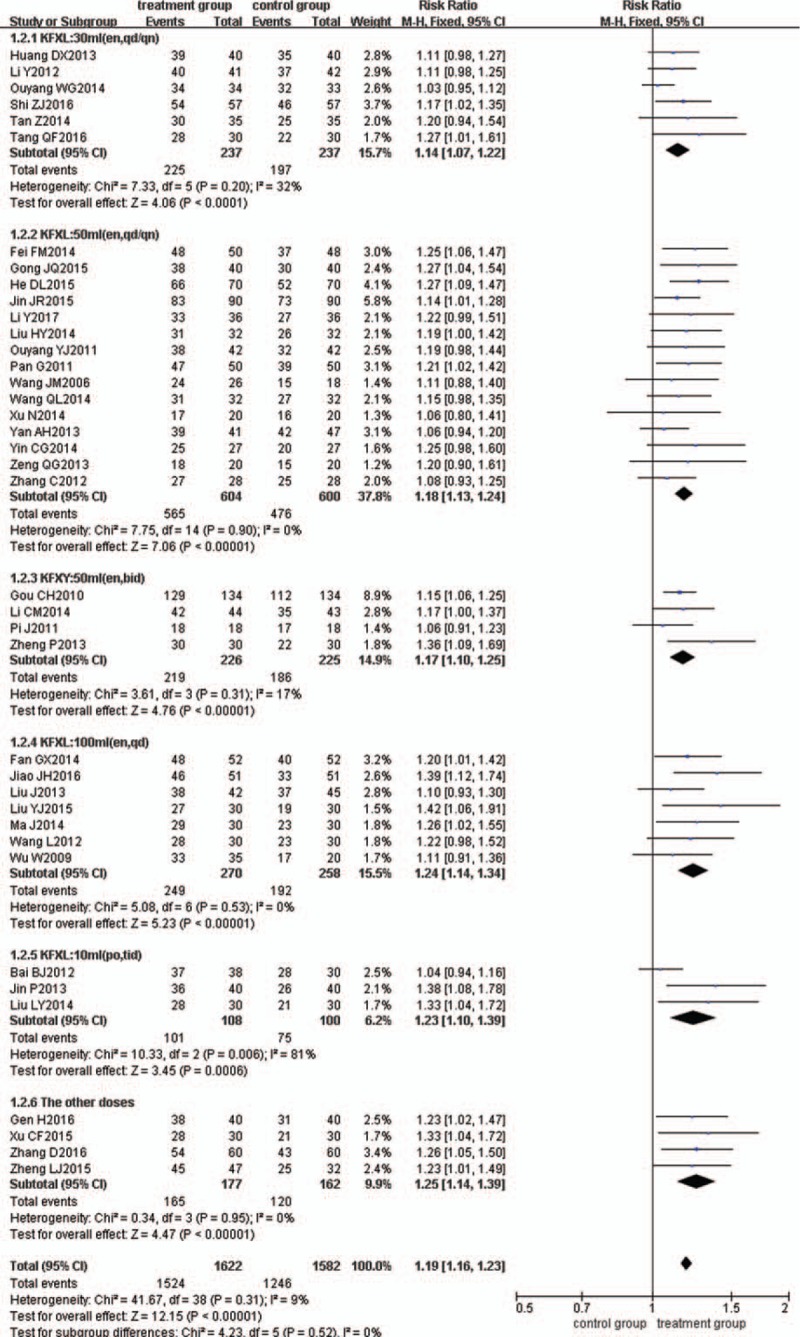
Meta-analysis of the clinical remission rate for the subgroup analysis of different doses.

Subgroup analysis was used to evaluate the efficacy of different doses. Compared with ASA alone, doses of 30 mL (en, qd/qn), 50 mL (en, qd/qn), 50 mL (en, bid), 100 mL (en, qd), and 10 mL (po, tid) of KFXL combined with ASA all had significant improvements in clinical remission, with RR = 1.14 (95% CI = 1.07, 1.22, *n* = 6), RR = 1.18 (95% CI = 1.13, 1.24, *n* = 15), RR = 1.17 (95% CI = 1.10, 1.25, *n* = 4), RR = 1.24 (95% CI = 1.14, 1.34, n = 7), and RR = 1.23 (95% CI = 1.10, 1.39, n = 3), respectively (Fig. 5).

### 3.7 Improvement of Intestinal mucosa

**Figure 6:**
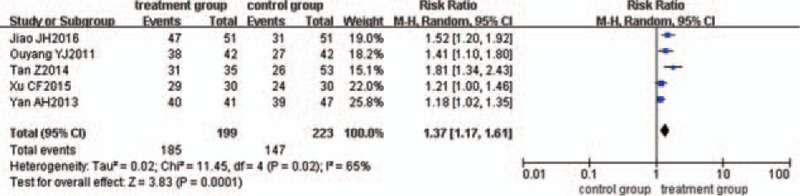
Meta-analysis of the curative effect of intestinal mucosa.

Five trials^[35,38,46,52,56]^ compared the improvement of intestinal mucosa. There was statistical heterogeneity between the 2 groups (*P = *.02, *I*^2^ = 65%), so the random-effects model was used. The pooled analysis revealed that the improvement of intestinal mucosa between the treatments was significantly different (RR = 1.37, 95% CI = 1.17, 1.61, *P = *.0001) (Fig. 6).

### 3.8 Reduction rate of UC symptoms

**Figure 7:**
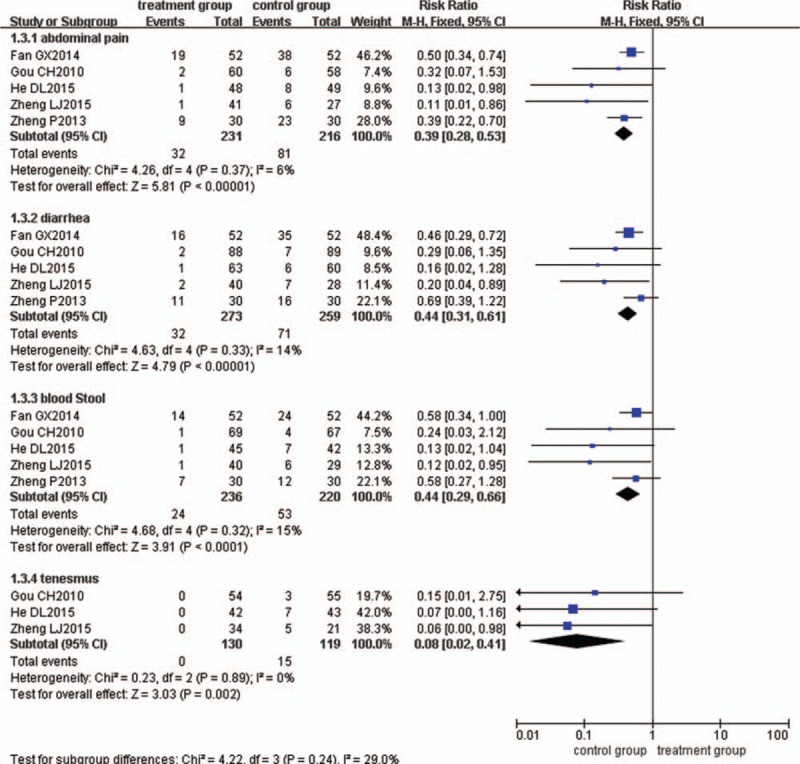
Meta-analysis of UC symptoms. UC = ulcerative colitis.

Five trials^[30,33,47,55,59]^ reported the reduction rate of UC symptoms. The meta-analysis results showed that in both the treatment and control group, there was a significant decrease in symptoms of abdominal pain, diarrhoea, bloody stool and tenesmus and that the reduction of UC symptoms in the control group was smaller than that in the treatment group (Fig. 7).

### 3.9 Abdominal pain

Five trials^[30,33,47,55,59]^ compared the abdominal pain between the 2 drug treatments. There was no significant heterogeneity between the 2 groups (*P = *.37, I^2^ = 6%), so the fixed-effects model was used. The pooled analysis suggested that the difference between the 2 groups was statistically significant (RR = 0.39, 95% CI = 0.28, 0.53, *P < *.00001) (Fig. 7).

### 3.10 Diarrhoea

Five trials^[30,33,47,55,59]^ compared the diarrhoea between the 2 drug treatments. There was no significant heterogeneity between the 2 groups (*P = *.33, *I*^2^ = 14%), so the fixed-effects model was used. The pooled analysis suggested that the difference between the 2 groups was statistically significant (RR = 0.44, 95% CI = 0.31, 0.61 *P < *.00001) (Fig. 7).

### 3.11 Bloody Stool

Five trials^[30,33,47,55,59]^ compared bloody stool between the 2 drug treatments. There was no significant heterogeneity between the 2 groups (*P = *.32, I^2^ = 15%), so the fixed-effects model was used. The pooled analysis suggested that the difference between the 2 groups was statistically significant (RR = 0.44, 95% CI = 0.29, 0.66, *P < *.0001) (Fig. 7).

### 3.12 Tenesmus

Three trials^[30,33,59]^ compared the tenesmus between the 2 drug treatments. There was no significant heterogeneity between the 2 groups (*P = *.89, I^2^ = 0%), so the fixed-effects model was used. The pooled analysis suggested that the difference between the 2 groups was statistically significant (RR = 0.08, 95% CI = 0.02, 0.41, *P = *.002) (Fig. 7).

### 3.13 Reduction of Inflammation factor Level

The inflammation factor level was evaluated in 9 trials.^[22–25,29,36,37,43,45]^ The number of trial participants ranged from 60 to 180. The meta-analysis results showed that the treatment groups were superior to the control groups in reducing the TNF-α, IL-1, IL-6, IL-8, and CRP levels.

### 3.14 Reduction of TNF-α

**Figure 8:**
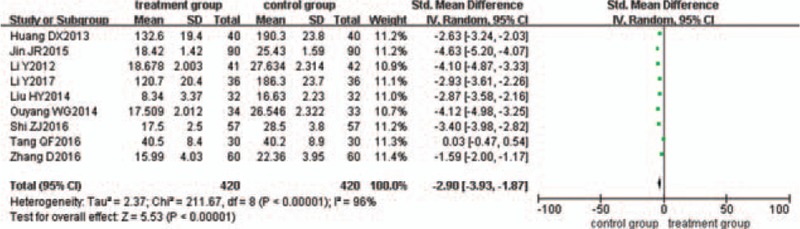
Meta-analysis of the reduction of TNF-αin ulcerative colitis.

Nine trials^[22–25,29,36,37,43,45]^ evaluated the effect of TNF-α reduction. There was statistical heterogeneity between the 2 groups (*P < *.00001, I^2^ = 96%), so the random-effects model was used. The pooled analysis suggested that the difference between the 2 groups was statistically significant (*P < *.00001; SMD = −2.90; 95% CI [−3.93, −1.87]) (Fig. 8).

### 3.15 Reduction of IL-1

**Figure 9:**
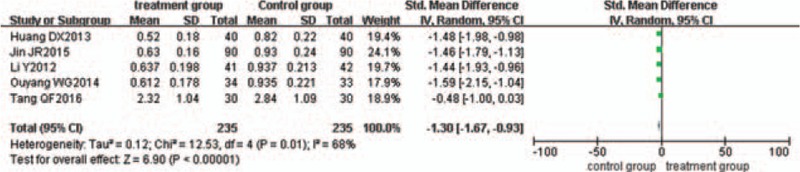
Meta-analysis of the reduction of IL-1 in ulcerative colitis.

Five trials^[22,23,29,36,37]^ evaluated the effect of IL-1 reduction. There was statistical heterogeneity between the 2 groups (*P = *.01, I^2^ = 68%), so the random-effects model was used. The pooled analysis suggested that the difference between the 2 groups was statistically significant (*P < *.00001; SMD = −1.30; 95% CI [−1.67, −0.93]) (Fig. 9).

### 3.16 Reduction of IL-6

**Figure 10:**
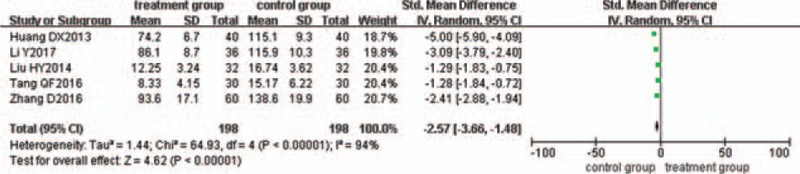
Meta-analysis of the reduction of IL-6 in ulcerative colitis.

Five trials^[22,23,25,43,45]^ evaluated the effect of IL-6 reduction. There was statistical heterogeneity between the 2 groups (*P < *.00001, I^2^ = 94%), so the random-effects model was used. The pooled analysis suggested that the difference between the 2 groups was statistically significant (*P < *.00001; SMD = −2.57; 95% CI [−3.66, −1.48]) (Fig. 10).

### 3.17 Reduction of IL-8

**Figure 11:**
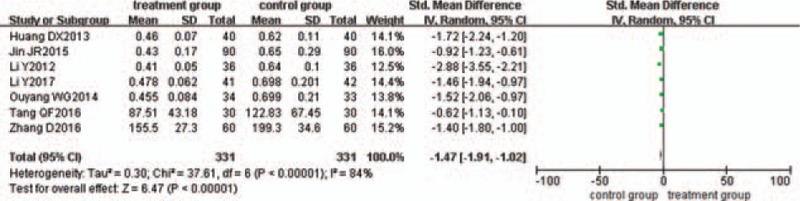
Meta-analysis of the reduction of IL-8 in ulcerative colitis.

Seven trials^[22,23,25,29,36,37,45]^ evaluated the effect of IL-8 reduction. There was statistical heterogeneity between the 2 groups (*P < *.00001, I^2^ = 84%), so the random-effects model was used. The pooled analysis suggested that the difference between the 2 groups was statistically significant (*P < *.00001; SMD = −1.47; 95% CI [−1.91, −1.02]) (Fig. 11).

### 3.18 Reduction of CRP

**Figure 12:**
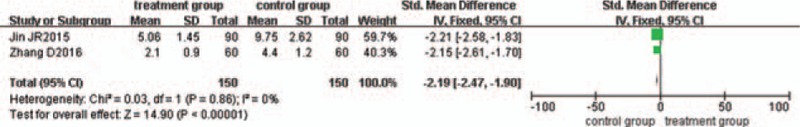
Meta-analysis of the reduction of CRP in ulcerative colitis. CRP = C-reactive protein.

Two trials^[29,45]^ evaluated the effect of CRP reduction. There was no significant heterogeneity between the 2 groups (*P = *.86, I^2^ = 0%), so the fixed-effects model was used. The pooled analysis suggested that the difference between the 2 groups was statistically significant (*P < *.00001; SMD = −2.19; 95% CI [−2.47, −1.90]) (Fig. 12).

### 3.19 Effect on coagulation index

The effect on coagulation index was evaluated in 3 trials.^[22,23,34]^ The meta-analysis results showed that the experimental groups were superior to the control groups in reducing the FIB and platelet (Plt) values and in increasing the PT and MPV values.

### 3.20 FIB

**Figure 13:**
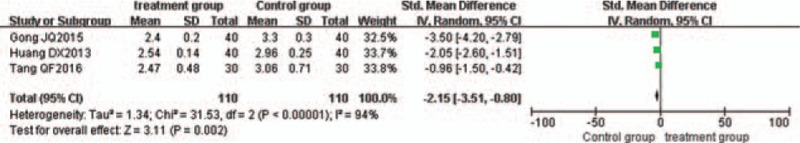
Meta-analysis of the reduction of FIB in ulcerative colitis. FIB = fibrinogen.

Three trials^[22,23,34]^ evaluated the effect of FIB reduction. There was statistical heterogeneity between the 2 groups (*P < *.00001, I^2^ = 94%), so the random-effects model was used. The pooled analysis suggested that the difference between the 2 groups was statistically significant (*P = *.002; SMD = −2.15; 95% CI [−3.51, −0.80]) (Fig. 13).

### 3.21 Plt

**Figure 14:**
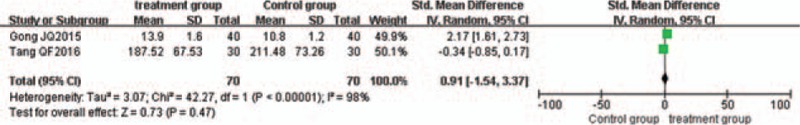
Meta-analysis of the reduction of Plt in ulcerative colitis. Plt = platelet.

Two trials^[23,34]^ evaluated the effect of Plt reduction. There was statistical heterogeneity between the 2 groups (*P < *.0001, I^2^ = 98%), so the random-effects model was used. The pooled analysis suggested that the difference between the 2 groups was not statistically significant (*P = *.47; SMD = 0.91; 95% CI [−1.54, 3.37]) (Fig. 14).

### 3.22 PT

**Figure 15:**
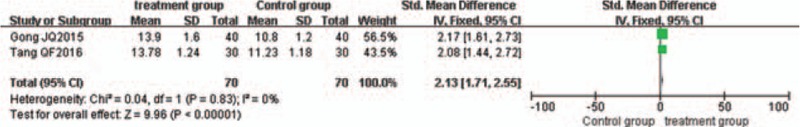
Meta-analysis of the increase of PT in ulcerative colitis. PT = prothrombin time.

Two trials^[23,34]^ evaluated the effect of PT increase. There was no significant heterogeneity between the 2 groups (*P = *.83, I^2^ = 0%), so the fixed-effects model was used. The pooled analysis suggested that the difference between the 2 groups was statistically significant (*P < *.00001; SMD = 2.13; 95% CI [1.71, 2.55]) (Fig. 15).

### 3.23 MPV

**Figure 16:**
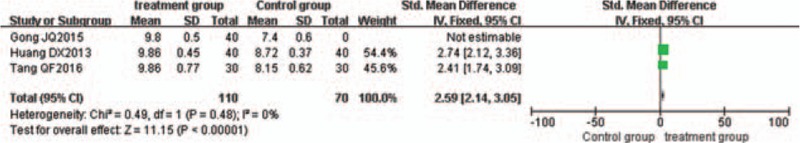
Meta-analysis of the increase of MPV in ulcerative colitis. MPV = mean platelet volume.

Three trials^[22,23,34]^ evaluated the effect of MPV increase. There was no significant heterogeneity between the 2 groups (*P = *.48, I^2^ = 0%), so the fixed-effects model was used. The pooled analysis suggested that the difference between the 2 groups was statistically significant (*P < *.00001; SMD = 2.59; 95% CI [2.14, 3.05]). The effect estimates are shown in Figure 16.

### 3.24 Relapse rate

**Figure 17:**
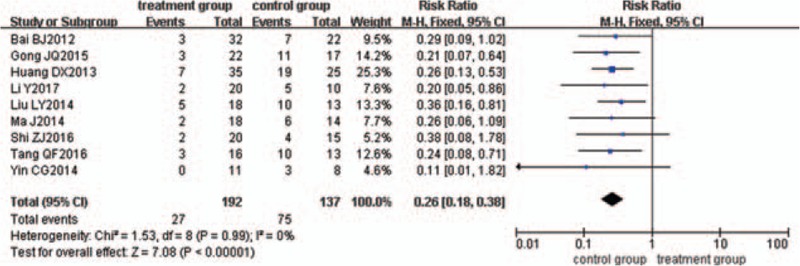
Meta-analysis of relapse rate.

Nine trials^[22–25,31,34,40,49,57]^ evaluated the effect of relapse rate. A total of 102 patients relapsed, with a rate of 27/192 in the treatment group and 75/137 in the control group. There was no significant heterogeneity for relapse rate between the 2 groups (*P = *.99, *I*^2^ = 0%). The meta-analysis was performed using a fixed-effects model. The pooled analysis suggested that the difference between the 2 groups was statistically significant (*P < *.00001), with RR of 0.26 and 95% CI (0.18, 0.38) (Fig. 17).

### 3.25 Adverse effects

**Figure 18:**
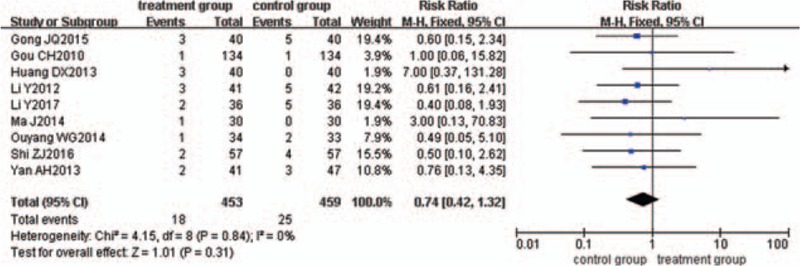
Meta-analysis of adverse reactions.

Through a careful reading of the 39 included studies, 14 trials^[22–25,27,34–40,56,59]^ mentioned the occurrence of adverse effects. Five trials^[23,27,35,38,39]^ reported that no adverse effects occurred. Nine trials^[22,24,25,34,36,37,40,56,59]^ reported adverse effects incidence; specifically, 18 out of 453 patients undergoing treatment with KFXL combined with ASA reported adverse events, and 25 out of 459 patients undergoing treatment with ASA showed side effects. The adverse events mainly included nausea, bloating, rash, headache, dizziness, and vomiting. No severe adverse events were reported. The remaining 25 trials^[26,28–33,41–55,57,58,60]^ did not mention the occurrence of adverse effects. The pooled analysis indicated that there was no obvious difference in the incidence of adverse effects between the 2 groups (*P = *.31), with RR of 0.74 and 95% CI (0.42, 1.32) (Fig. 18). The results suggested that KFXL combined with ASA might be a safe approach in managing UC.

### 3.26 Sensitivity analysis

To evaluate the reliability of our meta-analytical data, we tested sensitivity using the ‘leave-one-out’ approach. Removal of any one study from the analysis of clinical remission rate in UC patients did not significantly affect the outcome. Regardless of the exclusion of individual studies, the consistency in the direction and magnitude of the combined estimates indicated that the meta-analysis had good reliability.

### 3.27 Publication bias

**Figure 19:**
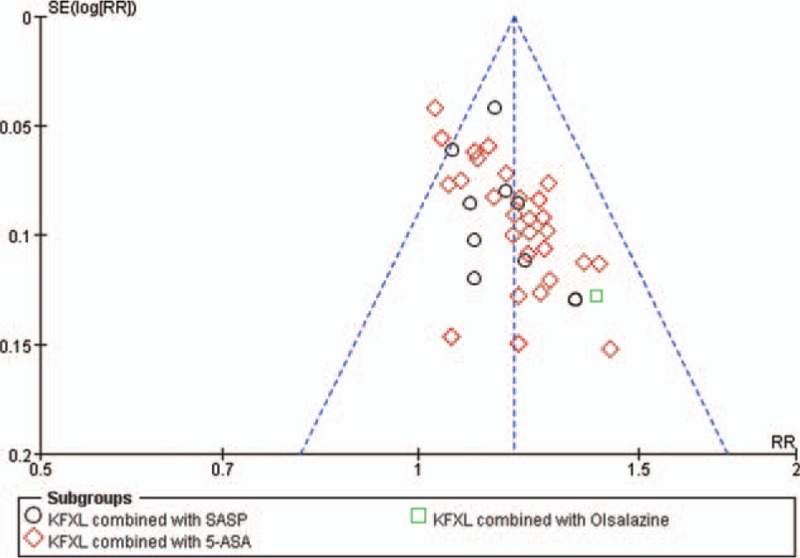
Funnel plot of clinical efficiency.

A forest plot of the comparison of KFXL combined with ASA and ASA alone for the outcome of clinical remission rates is shown in Figure 18. This test found significant evidence of publication bias for clinical remission rates in the studies (Fig. 19).

## 4 Discussion

### 4.1 Summary of evidence

This meta-analysis provides a quantitative synthesis of the clinical efficacy of KFXL combined with ASA for the treatment of UC by integrating outcomes from 39 clinical trials involving 3204 participants. In our study, twenty-8 trials are about KFXL combined with 5-ASA vs 5-ASA alone, ten trials are about KFXL combined with SASP vs SASP alone, and one trial is about KFXL combined with OSLS vs OSLS alone. The results from the meta-analysis revealed the following: compared to ASA used alone, KFXL combined with ASA treatment showed a higher clinical remission rate (RR = 1.19) and a lower relapse rate (RR = 0.26); compared with ASA alone, doses of 30 mL (en, qd/qn), 50 mL (en, qd/qn), 50 mL (en, bid), 100 mL (en, qd), and 10 mL (po, tid) of KFXL combined with ASA all significantly improved the clinical remission, with RR = 1.14 (95% CI = 1.07, 1.22, *n* = 6), RR = 1.18 (95% CI = 1.13, 1.24, n = 15), RR = 1.17 (95% CI = 1.10, 1.25, n = 4), RR = 1.24 (95% CI = 1.14, 1.34, n = 7), and RR = 1.23 (95% CI = 1.10, 1.39, n = 3), respectively; KFXL combined with ASA could significantly reduce the inflammation factor level of TNF-α, IL-1, IL-6, IL-8, and CRP in patients with UC; KFXL combined with ASA could improve the intestinal mucosa and symptoms in patients with UC; KFXL combined with ASA was superior to the control groups regarding reducing the FIB and Plt values and increasing the PT and MPV values; and compared to the control groups, KFXL combined with ASA showed a lower adverse effects rate, but the difference between the 2 groups was not statistically significant (*P = *.31). However, the overall estimated results should be interpreted cautiously, considering the high risk of bias.

### 4.2 Limitations

Certain limitations of our meta-analysis should be described. First, although we have conducted a comprehensive literature search in the 7 electronic databases, databases published in other languages except Chinese and English were not included in our study. All of the 39 included studies were conducted in China and published in Chinese; thus, some relevant publications of KFXL combined with ASA in treating UC might have been missed.

Second, only 14 out of 39 trials mentioned the occurrence of adverse effects. Among these, 5 trials reported that no adverse effects occurred, and 9 trials reported the adverse effects incidence. The rest of the included studies did not mention adverse events at all. So the safety of KFXL combined with ASA in the treatment of UC is limited. More trials are necessary to be conducted to assess the safety of KFXL combined with ASA in treating UC.

Third, we understand that negative results are often difficult to report in China, and all of the included studies reported positive results, so a certain degree of potential selection bias might exist. Previously published systematic reviews of Chinese herbal medicine have confronted similar questions.^[61,62]^

Fourth, the methodological quality in the studies was generally poor. All of the eligible articles were nonblinded RCTs. Though randomization was mentioned in all trials, only eleven trials reported the methods of randomization, and no trials reported the blinding of outcome assessment, the loss of cases, or intention analysis. Blinding and allocation concealment were not reported in these RCTs, which meant potential risk of implementation bias. These potential biases were more likely to overestimate the combined effect size. Further well-designed, randomized, double-blinded, multi-center studies are needed to make a more definite conclusion.

## 5 Conclusions

In conclusion, KFXL combined with ASA could improve clinical remission, symptoms, intestinal mucosa, inflammation factor level, coagulation index, and relapse rate in UC patients. This systematic review and meta-analysis provides an evidence-based approach to the management of UC. KFXL combined with ASA may be a new treatment for UC. However, some limitations such as potential selection bias and methodological flaws might undermine the validity of positive findings. From a clinical point of view, further RCTs with high-quality and long-term follow-up are recommended to generate a high level of clinical evidence.

## Acknowledgments

We thank the National Natural Science Foundation of China and Guangzhou University of Chinese Medicine for their funds.

## Author contributions

**Data curation:** De-tang Li.

**Formal analysis:** Zhen-wen Qiu.

**Methodology:** Hong-mei Tang.

**Writing – original draft:** Hui-biao Li, Mu-yuan Chen.

**Writing – review & editing:** Qing-qun Cai, Hong-mei Tang, Xin-lin Chen.

## Supplementary Material

Supplemental Digital Content
